# Fish sharing as a risk factor for *Opisthorchis viverrini* infection: evidence from two villages in north-eastern Thailand

**DOI:** 10.1186/s40249-017-0281-7

**Published:** 2017-04-04

**Authors:** Parichat Saenna, Cameron Hurst, Pierre Echaubard, Bruce A. Wilcox, Banchob Sripa

**Affiliations:** 1 WHO Collaborating Centre for Research and Control of Opisthorchiasis (Southeast Asian Liver Fluke Disease), Tropical Disease Research Laboratory, Department of Pathology, Faculty of Medicine, Khon Kaen University, 123 Mittraparb Road, Khon Kaen, 40002 Thailand; 2 Biostatistics Center of Excellence, Faculty of Medicine, Chulalongkorn University, Bangkok, 10330 Thailand; 3 Global Health Asia, Integrative Research and Education Program, Faculty of Public Health, Mahidol University, Bangkok, Thailand; 4 Department of Biology, Laurentian University, 935 Ramsey Lake Road, Sudbury, P3E2C6 ON Canada; 5 Present Address: Faculty of Education, Khon Kaen University, Khon Kaen, Thailand

**Keywords:** Foodborne trematodes, *Opisthorchis viverrini*, Opisthorchiasis prevention and control, Food sharing, Raw fish dishes, Social network analysis, Thailand

## Abstract

**Background:**

Foodborne trematodiasis (FBT) is a significant global health problem, with the liver flukes *Opisthorchis viverrini*, *O. felineus*, and *Clonorchis sinensis* contributing to half of the global burden of FBT. North-eastern Thailand where *O. viverrini* is endemic and un-cooked fish dishes remain an integral part of the food culture has the highest reported incidence of opisthorchiasis, including associated cholangiocarcinoma. Both food sharing and eating practices are potentially important factors in FTB, suggesting an important role for the social ecology of disease transmission in these rural communities.

**Methods:**

Two rural Thai-Lao villages that were part of a 12-village project in Northeastern Thailand were selected for detailed investigation of *O. viverrini* infection risk associated with sharing of raw fish dishes among households. The project included screening individuals for infection and cholangiocarcinoma, a household questionnaire, and offering treatment options for positive individuals. Social network mapping was used to construct raw fish dish-sharing networks and create a proxy variable capturing variability in the degree of food sharing (DFS), measured as the number of different households with which each household shared fish dishes. Measures of associations between DFS, *O. viverrini* infection, the frequency of raw fish consumption, and the number of raw fish dishes consumed were generated using binary logistic regression, proportional odds ordinal logistic regression, and Poisson regression.

**Results:**

The results showed that the probability that a household has members infected with *O. viverrini* increased by ~7% (*P* < 0.01) for each additional household included in its network. Moreover, the frequency and number of types of raw fish dishes consumed increased significantly as the DFS increased. Of the two villages, that with the highest infection prevalence (48% versus 34.6%) had significantly higher social connectivity overall (*P* < 0.001).

**Conclusions:**

Our findings suggest that the social ecology of human settlements may be key to understanding the transmission dynamics of some FBT. In the case of *O. viverrini* in Thai-Lao communities, for which food sharing is a traditional practice supporting social cohesion, food sharing network mapping should be incorporated into community-based interventions. These should encourage fish dish preparation methods that minimize infection risk by targeting households with high DFS values.

**Electronic supplementary material:**

The online version of this article (doi:10.1186/s40249-017-0281-7) contains supplementary material, which is available to authorized users.

## Multilingual abstracts

Please see Additional file 1 for translations of the abstract into the five official working languages of the United Nations.

## Background

Foodborne trematodiasis (FBT) infections have recently been described as an important cluster of neglected diseases. Conservative estimates conclude that more than 50 million people are infected worldwide [1]. Fürst et al. [1] estimated that slightly more than half of the FBT burden is due to the liver flukes *Opisthorchis viverrini*, *O. felineus*, and *Clonorchis sinensis*. It has long been known that liver fluke infections are caused by the consumption of raw freshwater fish, especially species of carp or minnow (*Family Cyprinidae*) [2]. Infection caused by *O. viverrini*, responsible for an estimated 70% of all human liver fluke infections in Southeast Asia [3], is considered to be one of the most clinically important trematode infections [4]. While most infected individuals are asymptomatic, high-intensity chronic infections along with other dietary risk factors, alcohol consumption, and smoking are associated with the development of cholangiocarcinoma (CCA) [5], which has a high fatality rate [3, 5].

Liver fluke control programs began in Thailand in the 1950s, with a national control program established in 1987 [6]. Since then, *O. viverrini* infection prevalence and CCA incidence have declined at the national level [5]. However, prevalence remains high in Northeastern Thailand, especially in Khon Kaen Province, where infection prevalence exceeding 50% at the village level is still commonly found [7, 8]. Moreover, Khon Kaen Province has the highest reported incidence of *O. viverrini* infection-associated CCA in the world [5].

This province and the surrounding area is at the geographic centre of the Lower Mekong Basin where the practice of gathering, preparing, and sharing of fish dishes is deeply rooted in the local culture. A more in-depth understanding of the attitudes and practices associated with raw-fish-eating practices are therefore needed [9]. These should be linked to participatory health education initiatives [10], ideally as part of an integrated, bottom-up control approach [8]. Indeed, recent advances in health-risk behaviour change theory, much of it based on the social ecological model [11], support this view [12]. The role of social ecology in foodborne parasite transmission has been identified as a major knowledge gap for helminthes [13] and should be further advocated to become part of a relevant framework for understanding disease transmission, as well as to delineate sustainable control strategies [8].

Interdependency and reciprocal reliance among households whose livelihoods depend on prevailing environmental constraints of poor soils, unpredictable rainfall, and drought [14, 15] have historically characterized north-eastern Thailand’s social ecology [16]. This and other unique aspects of village life in Isan, the name of the north-eastern region of Thailand which borders Laos and Cambodia, are well described in the famous biography *A child of the Northeast* by K. Boontawee [17]. Although Isan village life has been rapidly modernizing in recent decades, practices involving traditional foods obtained from the local environment tend to continue as a component of cultural identity [18]. This includes the sharing of raw fish dishes among households.

In this paper, we report on the world first ever study of raw fish sharing in rural villages in Thailand, its possible contribution to *O. viverrini* transmission among individuals as well as to infection prevalence in the population, and implications of the findings for future research and intervention approaches.

We apply a novel approach to FBT research based on graphical methods associated with social network analysis (SNA) combined with statistical analysis. In SNA, individual entities of a network, such as people or other ‘actors’, called ‘nodes’, are linked by relationships often called ‘ties’ in SNA nomenclature. Nodes and ties, which in this study are households and food-sharing relationships, can be readily mapped. The nodes are represented as points (or dots that can be sized according to a node’s importance in the network), while the ties are represented as lines. The lines can be of various types or colours to characterize the food-sharing relationship. The resulting graphic or map is a particularly effective tool for visually analysing a social or other kind network. An extensive theory and terminology associated with SNA and social structure research has been developed. However, in this paper, we attempt to show that simply mapping food-sharing relationships among households, combined with statistical modelling, may yield useful insights for understanding parasite transmission and disease risk. This information can, in turn, provide a basis for more effective public health interventions.

## Methods

### Study area and population

The study was conducted in Kosum Phisai District, Maha Sarakham Province, north-eastern Thailand, which is located 35 km southeast of Khon Kaen’s municipal boundary and 50 km northwest of Maha Sarakham Municipality. The ethnicity of the district’s population is Thai-Lao, who speak Isan-Lao, a dialect of Lao.

The area is a typical lowland floodplain along the Chi River, a major tributary of the Lower Mekong. The landscape consists of clusters of houses, surrounded by rice paddies, reservoirs, and patches of remnant native forest vegetation. Besides the river, the water source for crop irrigation, each village typically has a reservoir, which is used for storage of irrigated water and for household use, as well as for cultivating wild fish from the river. Opisthorchiasis tends to be endemic in such settings in contrast to areas which are more distant from rivers or other large water bodies, including reservoirs [19].

**Fig. 1:**
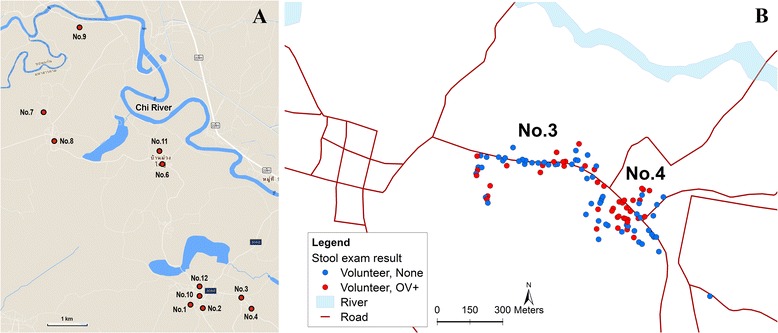
Geographical locations of the study sites. **a** Stool samples were collected and examined for the presence of *O. viverrini* eggs from participants in 12 villages (as indicated by the *red dots*) in Maha Sarakham Province, north-eastern Thailand. **b** This shows the spatial arrangement of households in villages 3 and 4. Households with members who are infected with *O. viverrini* are indicated by a *red dot*, while households free of infection are indicated by a *blue dot*. The satellite image was retrieved from Google Earth

The sampled population consisted of volunteer households located in the 12 rural villages in the Phon Ngam sub-district, located along the Chi River (see Fig. 1a).

### Study design and subsampling of villages

**Fig. 2:**
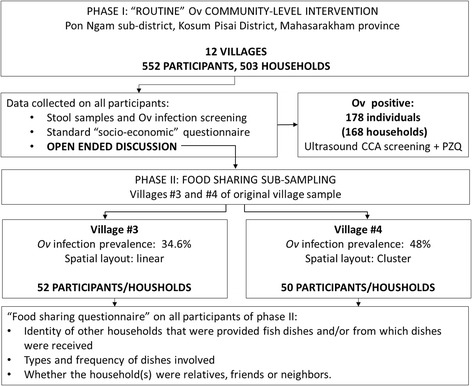
Study sequence and sampling characteristics

The project was initially designed as a routine, community-level intervention, at the sub-district level, which in this case consisted of 12 villages. It began with individuals who volunteered to participate by initially providing stool samples that were to be screened for *O. viverrini* infection. This was followed by all participants being interviewed using a brief questionnaire, ultrasound screening for CCA for those participants who were positive for *O. viverrini* infection, followed by appropriate medical consultation and treatment options offered individuals found to be positive. However, the standard routine of public health volunteers reading the questions to the interviewees and filling in the answers was not followed in this study. Rather, the volunteers were encouraged to conduct less formal, conversational interviews that were more open-ended. This resulted in additional information being elucidated, which suggested that raw fish sharing among households might be more common among those individuals who were positive for *O. viverrini* infection. However, as this information was not a questionnaire item and was gathered in conversation, unsystematically, it was not subject to statistical analysis (see Fig. 2).

Therefore, in order to investigate this finding further, two of the villages, villages numbered 3 and 4 of the 12, both with high *O. viverrini* prevalence (>30%), were selected for systematic study of the relationship between food sharing and *O. viverrini* infection. It was not possible to carry out this detailed investigation in more villages due to resource and logistical constraints, including the impending monsoon season that would render all but those two villages often inaccessible by road due to flooding. In addition, the proximity of villages 3 and 4 and their distinct spatial layout of households were considered an advantage. These villages represent two extremes: one village has homes closely clustered and in the other village the homes are more dispersed (usually linearly, with homes along a main road rather than in a grid plan). This is typically found in Thai villages in general and in the 12 villages comprising the study sub-district. We expected that the differences in the spatial arrangement of the households in the villages might entail different patterns of social interaction and food sharing. Thus, household arrangement could influence patterns of infection and explain the observed differences in infection prevalence (see Fig. 1b). We reasoned that a study of two villages representing each of the two extremes would provide a robust study design for testing the generality of any patterns of association between food sharing and *O. viverrini* infection. However, a more in-depth consideration of how the spatial arrangement of households may influence food sharing was beyond the scope of this study.

In order to visually characterize the spatial organization of the villages, household locations were determined using a handheld global positioning system receiver (Garmin Ltd.; Olathe, KS, USA), and household maps were then created using Quantum GIS version 1.8.0 (http://www.qgis.org/en/site/) based on this.

### Community engagement and data gathering

As is the case for most of the country, villages have public health volunteers who are recruited by government sub-district hospitals. The role of the public health volunteers is to facilitate government health interventions such as disease control campaigns. We worked with the Phon Ngam subdistrict hospital, which helped to coordinate our collaboration with public health volunteers from each of the 12 villages. The public health volunteers’ participation in the development of the questionnaire insured their solid understanding of the goals of the study and a relevant implementation of the research protocol. The public health volunteers’ role was also to distribute information to each household about our program, beginning with the offer of free examinations for liver fluke infection and free anti-helminthic medication for positive individuals. All public health volunteers were trained in stool sample collection and handling. Recruitment of participants was done through each village’s public address system as part of the daily announcements, in addition to public health volunteers visiting each household. Households that volunteered to participate, i.e., households with at least one member volunteering a stool sample along with their consent form, were interviewed using the questionnaire.

### Questionnaire development

The main questionnaire was formulated to gather information about the number of household members, and the participating household members’ gender, age, occupation, income, and frequency of eating raw fish dishes. The supplementary questionnaire used in the second phase (i.e. only including villages 3 and 4) consisted of questions solely about fish dish sharing in order to gather information about which households provided fish dishes and/or from which dishes were received, types of dishes consumed, and whether the household(s) were relatives, friends or neighbours.

The four types of raw fish dishes, each a well-known traditional staple of the Isan cuisine, are shown in Table 1. Each preparation uses one or more cyprinid fish species that are known to serve as a second intermediate host of *O. viverrini*, which thus makes them a source of potentially viable metacercariae [20].

**Table 1:** Type of raw, fermented, or partially cooked fish dishes regularly consumed in rural north-eastern Thailand

Local name of raw fish dish	Description
*Koi Pla* (ก้อยปลา)	Raw and spicy minced fish with herbs and lime juice
*Pla Som* (ปลาส้ม)	Short-term and sour fermented fish
*Pla Ra* (ปลาร้า)	Long-term fermented salty fish
*Pla Jom* (ปลาจ่อม)	Short-term fermented fish

The questions were developed and interviews were done in the Isan language, predominantly spoken by rural Isan-Lao people in the domestic environment. A pilot questionnaire was trialed and the wording of the questions was refined to ensure they were correctly understood both by the interviewers and interviewees. This was done for both the questionnaire used during the first phase (with all 12 villages) and the supplementary questionnaire used in the second phase (villages 3 and 4 only). The public health volunteers in each village conducted the interviews.

### Stool sample examination

Stool samples were preserved and examined for the presence of *O. viverrini* eggs immediately after collection. Preservation and examination were conducted by the Tropical Disease Research (TDR) Laboratory staff, Khon Kaen University, using the formalin-ether concentration method [21]. The sub-district hospital provided free-of-charge deworming medication (praziquantel) to those individuals who were found to be infected by *O. viverrini* (40 mg/kg; [22, 23]).

### Analysis of raw fish sharing and risk of *O. viverrini* infection

Statistical and visual analyses of raw fish sharing and *O. viverrini* infection risk associated with exposure to raw fish dishes were performed using the R statistical package version 3.0.3 (R Core Team, 2013).

Social network mapping was used to construct the raw-fish-sharing networks using the igraph R package [24]. The measure of association between *O. viverrini* infection and related risk factors with the degree of food sharing (DFS), defined as the number of households with which food is shared (regardless of whether the household was providing or receiving the food), were generated using one of several possible models, depending on the nature of the response variable being considered. Specifically, DFS was treated as a continuous predictor in all cases. Binary logistic regression was used to assess the association between DFS and *O. viverrini* infection status (i.e. a household with one or more positive members) and village (i.e. a proxy for the spatial arrangement of homes and hence the connectivity among households) (both binary variables). A proportional odds ordinal logistic regression was used to assess the association between DFS and the frequency of raw fish consumption (FC; i.e., the number of times per month raw fish dishes were consumed). A Poisson regression was computed to assess the association between DFS and the number of types of raw fish dishes (TD) (out of the four different dishes described in Table 1) consumed (represented by a count). For the logistic regression models, the measure of association produced was odds ratio (*OR*), while the rate ratio (*RR*) was generated for the Poisson regression, along with confidence intervals (*CI*s).

## Results

### Study population and sample households

In this study, 552 participants from 484 households (out of a total 503 households) in 12 villages submitted stool samples for examination. Out of these, 178 individuals (32.2%) from 168 households scored positive for *O. viverrini* infection. The prevalence of infection among the villages ranged from 12.3 to 48.0%.

The demographic characteristics of study participants from villages 3 and 4 are shown in Table 2. The majority of the participants (95%) were 40 years of age or older. This reflects the trend of urban migration and the disinterest of the younger adult household members in participating in the study (as they spent much of their time outside the villages during the times it was possible to gather data). Ninety-five out of 102 (93%) participants identified themselves as farmers. Locations of the participating households, including those with one or more persons who tested positive for *O. viverrini* infection, are shown in Fig. 1b. The consumption and sharing of raw fish and how these relate to the risk of acquiring *O. viverrini* infection among participants from villages 3 and 4 was further analyzed.

**Table 2:** Demographic characteristics of participants from villages 3 and 4

	Village no.		Total
	*3*	*4*	
Total participants (n)	52	50	102
*O. viverrini* prevalence (%)	34.6	48	41.3
Gender			
Male	29	29	58
Female	23	21	44
Occupation			
Farmer	46	49	95
Labourer	4	0	4
Unemployed	2	0	2
Other	0	1	1
Age (in years)			
< 20	0	0	0
20–39	1	4	5
40–59	30	16	46
≥ 60	21	30	51

### Sharing of raw fish dishes

**Fig. 3:**
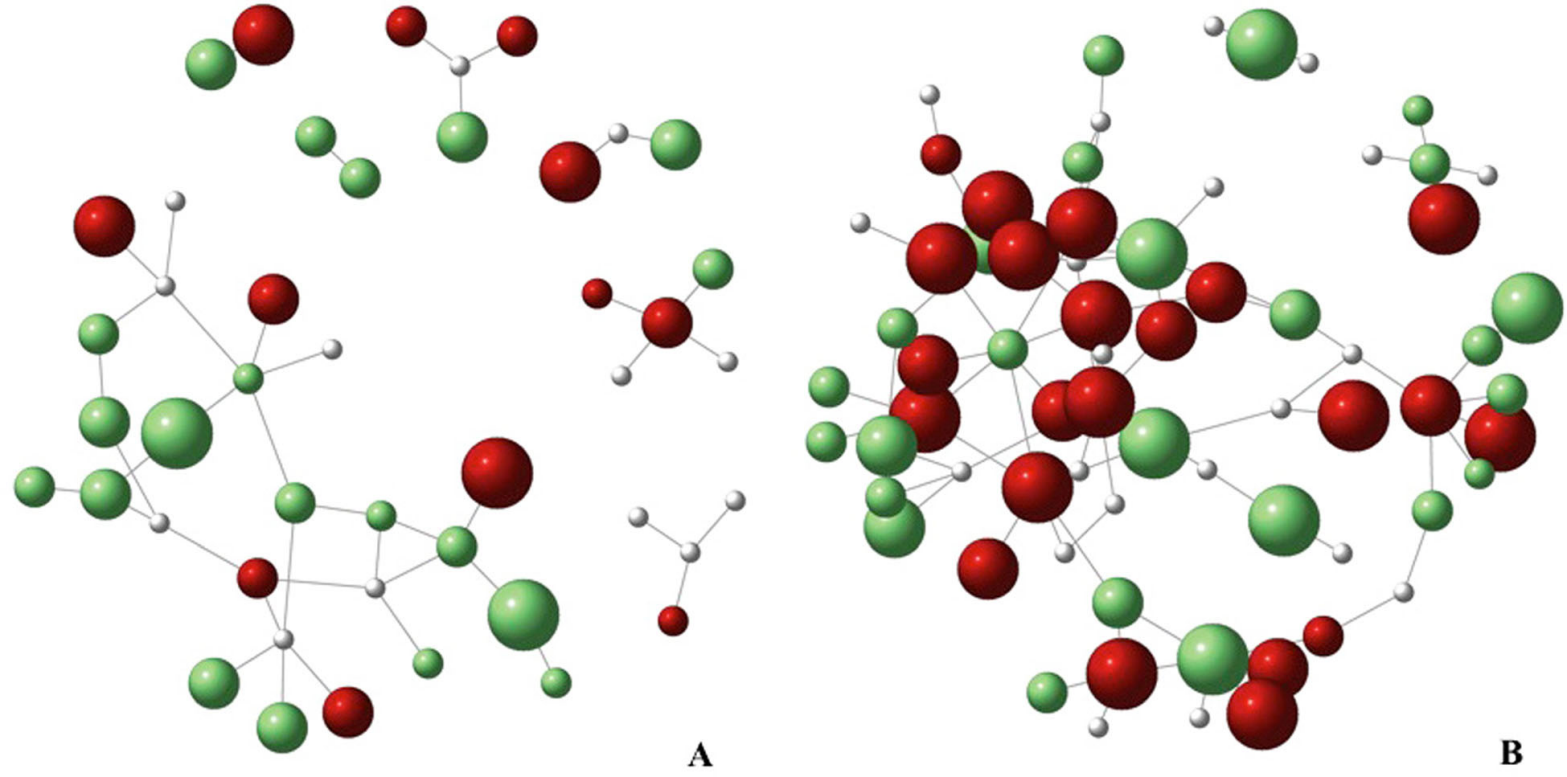
Raw-fish-sharing networks of villages 3 and 4. The graphs illustrate the patterns of raw fish sharing among households in village 3 (**a**) and 4 (**b**). *Red nodes* denote *O. viverrini*-infected households, *green nodes* denote households with members who tested negative for *O. viverrini* infection, and *white nodes* represent missing values. The size of the node reflects the number of raw fish dishes consumed in the household; bigger nodes represent higher raw fish dishes richness

DFS and FC varied considerably with respect to infection and TD, as shown in the network map (see Fig. 3). The raw-fish-dish-sharing network in village 4 (see Fig. 3b) is more complex (greater sharing connectivity) than that of village 3 (see Fig. 3a), with an apparent association observed between household food sharing and infection prevalence. However, households with members who were positive for *O. viverrini* infection also tended to consume a greater variety of raw fish dishes; that is, have a larger TD value (as indicated by the node size in the network maps).

With regard to the social determinants of raw fish dish sharing (who receives versus who provides fish dishes, which dishes, and at what frequency), we generally observed (although did not attempt to measure) that these behaviours largely revolve around three factors: shared expressed enjoyment of raw fish dishes in general or particular dishes by members of different households; male fisher groups who share fish dishes typically accompanied by alcohol outside (in shelters adjacent to the rice paddies, for example); and access to the types of fish used in the preparation of the raw fish dishes. Thus, households that did not have family members who fished and those consisting of elderly or otherwise disadvantaged individuals were typically recipients, while households that have members who fished and prepared fish dishes were typically providers.

### Estimation of infection risk and causality

To gain further insight into how, mechanistically, raw fish sharing and *O. viverrini* infection are linked, the associations between sharing and other risk factors, i.e. FC, number of TD, and village, as the variables, were analysed. The results are shown in Table 3.

**Table 3:** Crude association of DFS with *O. viverrini* infection and other *O. viverrini* infection risk factors

Outcome	Ratio	L95	U95
*O. viverrini* infection	1.33^a**^	1.06	1.65
Frequency of raw fish consumption	1.25^b*^	1.03	1.52
Types of consumed raw fish dishes	1.08^c**^	1.02	1.13
Village	1.60^a***^	1.23	2.07

^a^ *OR* from a binary logistic regression

^b^ *OR* from an ordinal logistic regression

^c^ *RR* from a Poisson regression

^***^ *P* < 0.001, ^**^ *P* < 0.01, ^*^ *P* < 0.05

A statistically significant association was found between DFS and the probability of *O. viverrini* infection (*χ* ^2^ = 6.98, degrees of freedom, df = 1, *P* < 0.01). For every additional household that a house shared raw fish with, there was a 33% increase in the odds of *O. viverrini* infection occurring (*OR* = 1.33, 95%*CI*: 1.06, 1.65). FC was also associated with the number of households that dishes were shared with (*χ* ^2^ = 5.52, df = 1, *P* < 0.05). The odds of going up to the next level of frequency raw fish consumption was 25% higher with each additional house that a dish was shared with (*OR* = 1.25, 95%*CI*: 1.03, 1.52). TD (1–4) was also associated with the degree of household sharing (*χ* ^2^ = 7.96, df = 1, *P* < 0.01). The probability of consuming a type of raw fish dish (i.e. eating one extra type of fish) increased by 8% with each additional household that a raw fish dish was shared with (*RR* = 1.08, 95%*CI*: 1.02, 1.13). There was also a difference in the degree of sharing between the two villages (*χ* ^2^ = 15.98, df = 1, *P* < 0.001).

## Discussion

It is known that human eating behaviour is influenced by a variety of factors including ethnicity, culture, religion, age, and gender [25]. In subsistence farmer and hunter-gatherer societies, the combination of which historically characterize the Isan livelihood [26], what is eaten and how is fundamentally a response to the local environment and its available resources [27]. Who eats together and how food is shared is basically a social ecological adaptation. What is eaten is determined by what is available, nutritious, and efficient to obtain or prepare. For rural farmers in north-eastern Thailand, besides glutinous rice, fish from the rice fields and adjacent wetlands have been a staple protein source for generations. Sharing food is a common practice of traditional farming societies [28], as it insures reciprocity. For Isan people, whose cultural practices generally reflect a history of making a living in a particularly harsh and unpredictable environment, food sharing is symbolic of sharing their identity [29]. Particularly in these circumstances, the most prized and nutritionally valuable food for Isan people besides rice is raw fish, which is also perhaps the most commonly shared food. Moreover, people share what they eat and eat what is shared with them. This study clearly showed that clusters of households with members infected with a disease tend to align with patterns of food sharing in the community.

The household maps for both study villages suggest a non-random distribution of *O. viverrini* infection (see Fig. 1b). This is consistent with the ‘clumping’ of infection intensity found for *O. viverrini* [10] and well known to be the case for helminthes generally [30]. Our results suggest that a greater degree of food sharing, determined by the spatial arrangement of households in a village, may explain the spatial clustering of *O. viverrini*-positive cases, as well as the infection prevalence at the village level. These associations are illustrated by the sharing network maps of villages 3 and 4. For instance, the pattern of spatial arrangement (location) of households is different in the two villages. Village 4 has a denser, more clustered pattern, while village 3 has a more linear, fragmented pattern. We assume this latter pattern to be less amenable to food sharing due to the greater physical distances between the households. This implies that there is less opportunity for frequent interaction, resulting in weaker social ties (all else being equal) and a need to invest more time for transit in order to either deliver or procure food. The higher infection prevalence observed in village 4 is consistent with the visual comparison of the two villages’ household spatial patterns and the sharing patterns shown by the network graphs. This also corroborates the positive and significant association between the degrees of sharing and infection prevalence, as suggested by our statistical model’s output.

The strong association between DFS, the number of households with which raw fish dishes are shared, and the household infection status can be explained by two related mechanisms and their nested associations. Food sharing is strongly and positively related to FC the frequency of raw fish dish consumption, and TD, the number of types of dishes, consumed. This means that households with a greater tendency to share food are most likely to receive, offer, and consume raw fish dishes as well. This, in turn, increases their risk of infection as compared to households that do not share food or do so only to a limited extent. That shared dishes tend to be mostly those made with raw fish corroborates other recent findings [9]. Smaller, less marketable, potentially infected *pla khao noi* (Isan-Lao for small cyprinid fish) that are mostly used in raw fish preparation are typically found in local villages for cheap sale or free distribution to family and friends of the fishermen (Kim et al. personal communication). Similarly, households also have a tendency to consume a greater diversity of raw fish dishes when they share with and receive food from others. As the number of sharing partners increases, so does the potential to receive different dishes made of different cyprinid fish species, which are competent hosts for *O. viverrini* metacercariae. These fish are known to present different patterns of metacercariae infection ([31], reviewed in [32]). Therefore, not every raw fish dish (i.e. comprised of different species) is similarly infectious. Yet, increasing the diversity of the types of raw fish shared and consumed increases the probability of being exposed to *O. viverrini*.

It is further worth noting that while the younger generation’s preference for modern Western food suggests a reduced consumption of traditional dishes and a de facto reduction of food sharing (as raw fish consumption is paired with the action of sharing it), raw fish consumption and food sharing remain a strongly engrained behaviour in individuals aged above 40 years (Kim et al., personal communication), including elderly people (>60 years). However, this generational difference should have had little effect on the results of this study, including the between-village network and infection prevalence differences, as the household samples had a similar number of individuals belonging to the 40–59- and >60-year-old cohorts.

Finally, we wish to emphasize that given the inherent limitations of our study sampling protocol (i.e. the households/individuals who participated in this study were recruited voluntarily and infection patterns observed may not accurately reflect infection distribution at the community level), firm conclusions about causal relationships between food sharing and prevalence of *O. viverrini* infection and CCA incidence require further investigation.

## Conclusions

Raw fish sharing among households in the rural villages of north-eastern Thailand may be an important factor contributing to *O. viverrini* infection and transmission. Greater connectivity among households increases the types of raw fish consumed as well as the frequency of raw fish consumption, and consequently the risk of *O. viverrini* infection. Targeting households that practice raw fish sharing as part of community-based interventions that promote cooked (or more thoroughly fermented) fish dishes should be strongly considered as an integral component of future disease control efforts. However, more research is also needed to assess in detail how food sharing contributes to the intensity of *O. viverrini* infection per capita, which, from a clinical perspective, represents a much more significant risk factor than infection prevalence [33]. Additionally, a better understanding is needed about how interventions can reduce risky raw fish consumption without discouraging food-sharing behaviour that reinforces social coherence, a well-known positive health factor. Sharing and eating traditional foods is likely an important contributor to individuals’ sense of coherence (i.e. psychosocial dimensions of health; [34, 35]), as well as the social coherence of communities. Weighing this against the physiological risk of *O. viverrini* infection could contribute to interventions being better aligned with communities’ overall health development.

## Additional file

Additional file 1: Multilingual abstracts in the five official working languages of the United Nations. (PDF 772 kb)

## Abbreviations

CCA: Cholangiocarcinoma
*CI*: Confidence interval
df: degrees of freedom
DFS: Degree of food sharing
FBT: Foodborne trematodiasis
FC: Frequency of raw fish consumption
OR: Odds ratio
*RR*: Rate ratio
SNA: Social network analysis
TD: Types of raw fish dishes
TDR: TROPICAL Disease Research
