# Supplementary material for: Fish sharing as a risk factor for Opisthorchis viverrini infection: evidence from two villages in north-eastern Thailand
Source: Infect Dis Poverty. 2017 Apr 4;6:66. doi: 10.1186/s40249-017-0281-7 (PMC5379607; doi:10.1186/s40249-017-0281-7)
Supplement: Additional file 1: — Multilingual abstracts in the five official working languages of the United Nations. (PDF 772 kb) [file 40249_2017_281_MOESM1_ESM.pdf]

Translation of the abstract into the five official working languages of the United Nations

مشاركة الأسماك كعامل خطر للإصابة بعدوى متأخر الخصية الزبادي : أدلة من قريتين في شمال شرق تايلاند

باريشتا سيينا، كاميرون هيرست، بيير اكهاوبارد، بروس ا. ويلكوكس، بانشوب سريبا

الخلاصة:

المعلومات الأساسية: يعد داء المثقوبات المنتقل عن طريق الأغذية (FBT) مشكلة صحية عالمية كبيرة، حيث تساهم عدوى مثقبيات الكبد و متأخر الخصية بانواعه الزبادي و الهري، و الصيني إلى نصف العبء العالمي الناجم عن داء المثقوبات المنتقل عن طريق الأغذية. يتم تسجيل اعلى حالات الاصابة بعدوى متاخر الخصية و سرطانة الأوعية الصفراوية المصاحبة له في شمال شرق تايلاند حيث ان داء متأخر الخصية الزبادي متوطن هناك ولكون السمك النيء طبقاً شعبياً اساسياً هناك. طرق مشاركة وتناول الطعام يمكن أن تكون عوامل هامة للاصابة بداء المثقوبات المنتقل عن طريق الأغذية مما يدل على أنهنالك دور هام تلعبه البيئة الاجتماعية في انتقال المرض في مثل هذه المجتمعات الريفية.

الأساليب: اختيرت قريتان ريفيتان من اصل 12 قرية تايلاندية ولالوسية كانت جزءا من مشروع في شمال شرق تايلاند لكي يخضعن لدراسة تفصيلية عن خطر الإصابة بداء متأخر الخصية الزبادي والخطر الناجم عن تبادل أطباق الأسماك النيئة بين الأسر. وتضمن المشروع فحص الأفراد بحثا عن الاصابة بالعدوى و سرطانة الأوعية الصفراوية، وشمل استبيانا منزليا، و تقديم خيارات علاجية للأفراد المصابين. تم استخدام طريقة تعيين شبكات اجتماعية لايجاد الشبكات المشاركة لاطباق السمك النيء وتكوين عازل متغير لايجاد المتغيرات في درجة مشاركة الطعام, والتي تمثل بقياس عدد الاسر المختلفة التي تشارك كل عائلة معها اطباق السمك. تم استخدام طرق الانحدار اللوجستي الثنائي و الانحدار اللوجستي الترتيبي ذو الاحتمالية النسبية و انحدار بواسون لايجاد درجة الصلة بين درجة مشاركة الطعام وبين داء متأخر الخصية الزبادي, و وتيرة استهلاك الاسماك, و بين عدد الاطباق المستهلكة.

النتائج: أظهرت النتائج بأن احتمال وجود أفراد مصابين باداء متأخر الخصية الزبادي في الاسرة قد ارتفع بنسبة ~ 7٪ (P <0.01) لكل أسرة إضافية تم ادراجها في شبكتها. بالإضافة إلى ذلك، فإن وتيرة وعدد أنواع أطباق السمك النيء المستهلكة قد ازدادات بشكل ملحوظ مع زيادة درجة مشاركة الطعام. من بين القريتين، كانت القرية التي سجلت نسبة أعلى لانتشار العدوى (48٪ مقابل 34.6٪) ذات تواصل اجتماعي أعلى بكثير بين افرادها عموما (P <0.001).

الاستنتاجات: تشير نتائجنا إلى أن البيئة الاجتماعية للمستوطنات البشرية قد تكون لسبيل لفهم آليات انتقال داء المثقوبات المنتقل عن طريق الأغذية (FBT). في حالة داء متأخر الخصية الزبادي في المجتمعات التايلاندية اللاوسية، التي تمثل مشاركة الطعام فيها ممارسة تقليدية داعمة للتلاحم الاجتماعي، فينبغي هنا ادراج طريقة تعيين الشبكات الاجتماعية المشاركة للطعام ظمن الأنشطة المجتمعية. حيث ان هذا من شأنه ان يشجع على طرق إعداد اطباق اسماك يكون من دورها ان تقلل من خطر الإصابة عن طريق التركيز على الأسر ذات درجات مشاركة الطعام العالية.

Translated from English version into Arabic by Muthanna, through

TRANSLATORS
WITHOUT BORDERS

鱼肉分享是感染麝猫后睾吸虫的危险因素：来自泰国东北部 2 个村庄的证据

Parichat Saenna, Cameron Hurst, Pierre Echaubard, Bruce A. Wilcox, Banchob Sripa

摘要：

引言：食源性吸虫病是重要的全球卫生问题，其中麝猫后睾吸虫、猫后睾吸虫和华支睾吸虫等肝吸虫病占了全球食源性吸虫病疾病负担的一半。泰国东北部是麝猫后睾吸虫的流行区，享用不熟鱼肉是当地饮食文化的重要组成部分。那里是麝猫后睾吸虫病及相关胆管癌发病率报告最高的地区。食物分享和饮食行为均是食源性吸虫病潜在的重要因素，说明了这些农村地区社会生态学在疾病传播中的重要作用。

方法：在泰国东北部有一个涉及 12 个村庄的项目，从中选择了 2 个泰-老民族村对与麝猫后睾吸虫感染相关的家庭生鱼肉分享情况进行详细的调查。项目内容包括对个体感染和胆管癌的筛查、家庭问卷调查以及为感染者提供治疗。应用社会网络图构建生鱼肉分享网络并生成替代变量获取家庭食物分享度（degree of food sharing，DFS）的变异性。DFS 指不同家庭里每个家庭分享生鱼肉的数量。通过二变量 logistic 回归、成比例序列 logistic 回归和 Poisson 回归计算 DFS、麝猫后睾吸虫感染、食用生鱼肉频次和食用生鱼肉数量之间的关系。
结果：研究发现如果某一家庭中有成员感染麝猫后睾吸虫，那么网络中的其他家庭风险增加约 7%（$P < 0.01$）。而且，随着 DFS 增加，食用生鱼肉的频次和数量也显著增加。2 个调查村的感染率分别为48%和 34.6%，而高感染率的村总体上具有更高的社会连通性（$P < 0.001$）。
结论：本研究表明人类定居的社会生态学对于了解一些食源性吸虫病的传播动态学至关重要。以泰-老民族村的麝猫后睾吸虫为例，食物分享是该地区的传统，促进了社会的连通。因此需要将食物分享网络图整合进干预措施中，要在针对那些高 DFS 的家庭宣传鱼肉烹制方法以降低感染风险。

Translated from English version into Chinese by Men-Bao Qian, through

Le partage de poisson comme un facteur de risque d'infection en Opisthorchis viverrini: Preuve de deux villages du nord-est de la Thaïlande

Parichat Saenna, Cameron Hurst, Pierre Echaubard, Bruce A. Wilcox, Banchob Sripa

Sommaire

Contexte: Les trématodoses d'origine alimentaire posent un vrai problème de santé mondial, avec les douves du foie Opisthorchis viverrini, O. felineus et Clonorchis sinensis contribuant à la moitié du fardeau mondial des trématodoses. Au Nord-Est du Thaïlande où O. viverrini est endémique et les plats de poisson non cuits forment une partie intégrante de la culture alimentaire, il y a une plus forte incidence rapportée d'opisthorchiasis, y compris le cholangiocarcinome associé. Le partage des plats et les pratiques alimentaires sont potentiellement des facteurs importants dans les trématodoses, ce qui suggère un rôle significatif pour l'écologie sociale de la transmission de la maladie dans ces communautés rurales.
Méthodes: Deux villages ruraux Thai-Lao qui faisaient partie d'un projet de 12 villages dans le nord-est de Thaïlande ont été sélectionnés pour l'enquête détaillée du risque d'infection *en O. viverrini* associé au partage de plats de poissons crus entre les ménages. Le projet comprenait le dépistage de personnes pour l'infection et le cholangiocarcinome, un questionnaire sur les ménages et l'offre d'options de traitement pour les personnes séropositives. La cartographie du réseau social a été utilisée pour construire des réseaux de partage de plats de poisson cru et de créer un intermédiaire variable qui capture la variabilité dans le degré de partage des aliments, mesurée par le nombre de ménages avec lesquels chaque ménage a partagé des plats de poisson. Les mesures d'associations entre le degré de partage des aliments, l'infection en *O. viverrini*, la fréquence de la consommation de poisson cru, et le nombre de plats de poisson cru consommés ont été générés en utilisant une régression logistique binaire, une régression logistique de cotes proportionnelles ordinales, et une régression de Poisson.
Résultats: Les résultats ont montré que la probabilité qu'un ménage ait des membres infectés par O. viverrini a augmenté de ~ 7% (P <0,01) pour chaque ménage supplémentaire compris dans son

réseau. En outre, la fréquence et le nombre de types de plats crus de poissons consommés ont augmenté de manière significative avec l'augmentation de degré de partage des aliments. Sur les deux villages, celui avec la prévalence de l'infection la plus élevée (48% contre 34,6%) avait une connectivité sociale globale significativement plus élevée (P <0,001).
Conclusions: Nos résultats suggèrent que l'écologie sociale des établissements humains peut être la clé pour comprendre la dynamique de transmission de certaines trématodoses d'origine alimentaire. Dans le cas d'O. viverrini chez les communautés Thai-Lao, pour lesquels le partage des aliments est une pratique traditionnelle qui renforce la cohésion sociale, la cartographie du réseau de partage de la nourriture devrait être intégrée dans les interventions communautaires. Ceux-ci devraient encourager les méthodes de préparation de plat de poisson qui minimisent les risques d'infection en ciblant les ménages avec des valeurs élevées du degré de partage des aliments.

Translated from English version into French by CHAKIB ROULA, through

TRANSLATORS
WITHOUT BORDERS

Совместное использование рыбы в качестве фактора риска для инвазией opisthorchis viverrini инфекции: Доказательства из двух деревнях в северо-восточной части Таиланда

Parichat Saenna, Cameron Hurst, Pierre Echaubard, Bruce A. Wilcox, Banchob Sripa

Реферат
Фон: Trematodiasis пищевого происхождения (FBT) является серьезной проблемой глобального здравоохранения, с печени сосальщики инвазией opisthorchis viverrini, O. felineus и Clonorchis синенсис, способствующих половины глобального бремени ФБТ. На северо-восточном Таиланде, где O. viverrini является эндемиком и неприготовленные рыбные блюда остаются неотъемлемой частью культуры питания и имеет наибольшее число случаев описторхоза, в том числе связанные холангиокарциномы. Совместный прием пищи и образы приема пищи являются потенциально важными факторами в ФТБ, что указывает на важную роль для социальной экологии передачи инфекции в этих сельских общин.
Методы: Два сельских Тайско-Лаосской деревни, которые являются частью 12-село на северо-востоке Таиланда были отобраны для детального изучения O. viverrini риск инфекции, связанной с совместным использованием сырьевых рыбных блюд среди населения. Проект включал в себя скрининг лиц для инфекции и холангиокарцинома, вопросник для домохозяйств, и предлагает варианты лечения для инфицированных. Сопоставление социальной сети была использована построить сырое рыбное блюдо-сети и создать прокси-переменной захвата изменчивость в степени распределения продуктов питания (DFS), измеряемая как количество разных семей, с которыми каждая семья поделилась рыбными блюдами. Меры ассоциации между DFS, O. viverrini инфекции, частота потребления сырой рыбы, и количество употребляемых в сыром виде рыбные блюда были сделаны с использованием бинарной логистической регрессии, пропорциональные коэффициенты порядковой логистической регрессии и регрессии Пуассона.
Результаты: Результаты показали, что вероятность того, что члены семьи инфицированы O. viverrini увеличился на ~7% (p<0,01) за каждое дополнительное семейство включенное в его сеть. Кроме того, частота и количество видов потребляемого сырья рыбных блюд

значительно выросла, так как DFS увеличился. Из двух сел, что с высоким уровнем распространенности инфекции (48% против 34.6%) имели значительно более высокие социальные связи в целом ($p<0,001$).
Заключение: Наши результаты показывают, что социальная экология населенных пунктов может быть ключом к пониманию динамики передачи некоторых FBT. В случае O. viverrini в Тайско-Лаосской общины, для которых совместный прием пищи--это традиционная практика поддержки социальной сплоченности, картографирование сети совместного приема пищи должны быть включены в общин. Они должны поощрять рыбы способы приготовления блюд, которые сводят к минимуму риска заражения выявления домохозяйств с высокими значениями DFS.

Translated from English version into Russian by Hao-Qi Zhang.

Compartir el pescado como factor de riesgo de infección por *Opisthorchis viverrini:* Datos de dos poblados del noreste de Tailandia.

Parichat Saenna, Cameron Hurst, Pierre Echaubard, Bruce A. Wilcox, Banchob Sripa

Resumen
Antecedentes: La trematodiasis de transmisión alimentaria (TTA) es un problema sanitario global muy importante. Los trematodos hepáticos *Opisthorchis viverrini, O. felineus*, y *Clonorchis sinensis* contribuyen a la mitad de la carga mundial de TTA. El noreste de Tailandia, donde *O. viverrini* es endémico y los platos de pescado crudo siguen siendo una parte integrante de la cultura alimentaria, presenta la mayor incidencia de casos de opistorquiasis comunicados, que incluyen al colangiocarcinoma relacionado. Tanto la costumbre de compartir alimentos como la manera de comer son factores potencialmente importantes en la TTA, lo que sugiere el importante papel que tiene la ecología social de la transmisión de la enfermedad en esas comunidades rurales.
Métodos: Se seleccionaron dos poblados rurales Lao-Thai que formaron parte de un proyecto de 12 poblados del noreste de Tailandia para llevar a cabo una investigación minuciosa del riesgo de infección por *O. viverrini* infección vinculado a compartir platos de pescado crudo entre los hogares. El proyecto consistía en examinar a las personas para detectar infecciones y colangiocarcinoma, un cuestionario para el hogar, y ofrecer opciones de tratamiento para las personas que tuvieran resultados positivos. Se usó cartografía de redes sociales para elaborar redes de intercambio de platos de pescado y crear una variable indirecta para captar la variabilidad en el grado de intercambio de alimentos ("degree of food sharing", DFS), determinado como el número de hogares distintos con los que cada hogar compartía platos de pescado. Las mediciones de asociaciones entre el DFS, la infección de *O. viverrini*, la frecuencia de consumo de pescado crudo y el número de platos de pescado crudo consumidos se generaron mediante modelos de regresión logística binaria, regresión logística ordinal de probabilidad proporcional y regresión de Poisson.
Resultados: Los resultados mostraron que la probabilidad de que un hogar tuviera miembros infectados con *O. viverrini* aumentaba en ~7% ($P<0.01$) para cada hogar individual comprendido en su red. Además, la frecuencia y el número de tipos de platos de pescado crudo consumidos aumentaban significativamente a medida que aumentaba el DFS. De los dos poblados, el que presentaba la prevalencia mayor de infección (48% frente a 34.6%) presentaba en conjunto una conectividad social mucho mayor ($P<0.001$).
Conclusiones: Nuestros resultados sugieren que la ecología social de los asentamientos humanos puede ser fundamental para entender la dinámica de transmisión de algunas TTA. En el caso de *O. viverrini* en comunidades Lao-Thai, en donde compartir la comida es una práctica tradicional que apoya la cohesión social, la cartografía de redes sociales de intercambio de alimentos se debe

incorporar en las intervenciones en la comunidad. Esto debería propiciar métodos de preparación de platos de pescado que minimizaran el riesgo de infección apuntando a hogares con valores altos de DFS.

Translated from English version into Spanish by Beatriz Grucci, through

TRANSLATORS
WITHOUT BORDERS
